# A serious skin virus epidemic sweeping through the Indian subcontinent is a threat to the livelihood of farmers

**DOI:** 10.1080/21505594.2022.2141971

**Published:** 2022-11-01

**Authors:** Naveen Kumar, Bhupendra Nath Tripathi

**Affiliations:** National Centre for Veterinary Type Cultures, ICAR-National Research Centre on Equines, Hisar, India; Animal Science Division, Indian Council of Agricultural Research, Krishi Bhawan, New Delhi, India

**Keywords:** Lumpy skin disease, Indian subcontinent, epidemic, 2022, livelihood

Countries in the Indian subcontinent, including India, Pakistan, Bangladesh, Nepal, Sri Lanka and Bhutan, are currently facing a deadly viral epidemic in the cattle population. The disease is called lumpy skin disease (LSD), which is caused by a *Capripoxvirus* (family *Poxviridae*) called lumpy skin disease virus (LSDV). The disease is characterized by fever, enlargement of the lymph nodes, anorexia, depression, dysgalactia, emaciation and development of skin nodules (10–50 mm in size) that can lead to a sharp decline in milk production, abortion in pregnant cattle and sterility in bulls [1].

For many decades since its first report from Zambia (Africa) in 1929 [2], LSD was confined to the African countries. Since 2012, LSD has spread from Africa into several countries in Europe. In Asia, it was first reported in 2019 in NorthWest China, Bangladesh, and India [1,3,4] and then in 2020, it spreads across many countries, including Bhutan, Myanmar, Nepal, Hong Kong, Vietnam, Taiwan and Sri Lanka. In contrast to the vaccine-like LSDV strains circulating in China and Russia, the LSDV strains being reported in the Indian subcontinent since 2019 are similar to Kenyan-type LSDV strains [5].

For a period of about 2–3 years since its introduction into India, incidences of LSD were mainly observed in the Eastern part of the country without any significant mortality. The current wave of the LSD, which was started from the Western Indian states of Gujarat and Rajasthan in June/July 2022, is highly lethal. According to the Department of Animal Husbandry and Dairying, Government of India, as of 30 September 2022, LSD has spread across 251 districts in 15 states and has affected over 2 million animals including 100,000 deaths [6]. The milk production has decreased by more than 26% in the affected states [6]. If LSD continues, India and other countries in the region will face a major milk shortage. Livestock farming is the mainstay of the small and marginal farmers (>85% of the total farmers) in the subcontinent. The maximum number of deaths due to LSD has occurred in lactating and pregnant cattle, which serve as a direct source of income in terms of the sale of milk and produce (livestock), thereby adversely affecting the livelihood of poor farmers in the region. The direct livestock and production losses due to LSD in the Asian countries are estimated to be worth up to $1.46 billion [7].

In contrast to the recent LSD outbreaks, which are associated with high mortality, LSD is usually not associated with high mortality [8]. Although the precise molecular mechanism of high pathogenicity of LSDV/2022 strain currently circulating in India and other South Asian countries needs to be determined, our preliminary observations (post-mortem findings) suggest that high mortality may be due to the capacity of the virus (LSDV/2022) to cause severe haemorrhages and extensive nodule formation in the visceral organs, especially lungs.

LSD is primarily a disease of cattle. Buffaloes develop only mild illness [9]. Other domestic animals are considered to be resistant to LSDV infection [10]. Our laboratory has confirmed mild LSDV infection in camels, horses and deer, as evidenced by the presence of viral DNA in skin nodules and anti-LSDV antibodies in convalescent serum samples (unpublished). This suggests the capability of LSDV to infect other host species, and therefore, necessitates inclusion of these species in the epidemiology and control of LSDV infection in animals. There are growing concerns about the zoonotic implications of LSD, but confirmatory evidences of human infection are still lacking.

LSDV belongs to the genus *Capripoxvirus* of the family *Poxviridae*. Two other capripoxviruses, sheeppox virus (SPV) and goatpox virus (GPV), which cause similar diseases in sheep and goats, respectively, are genetically and antigenically quite similar and cannot be distinguished from each other serologically. Heterologous vaccines (based on SPV or GPV) are usually authorized for use to induce cross protection against LSDV in cattle, particularly in instances where a homologous (LSDV-based) vaccine is not available. The Government of India also authorized the use of a heterologous vaccine (GPV-based) against LSD in cattle. However, heterologous vaccines are less efficacious and only provide partial protection [11].

The National Centre for Veterinary Type Cultures in Hisar (India), in collaboration with the Indian Veterinary Research Institute in Izatnagar (India) developed a homologous live-attenuated LSD vaccine, named Lumpi-ProVac^Ind^. The virus used for developing the vaccine was isolated from skin scab collected from a naturally LSDV-infected cattle from Ranchi (India) in 2019 (Kenyan-type LSDV strain). The virus was attenuated by continuous cell culture passaging (50 times in Vero cells). The experimental vaccination trials in calves suggested that the vaccine was safe, could induce potent levels of antibody- and cell-mediated immune response and provided a complete level of protection against virulent LSDV infection in cattle. As part of phase III clinical trials, the vaccine has been administered in over 25,000 animals in different geographical regions in India. The vaccine has been found to be safe in cattle and buffaloes of all age groups including lactating/pregnant animals and bulls. Further, we have observed that LSDV/India/2022 can be completely neutralized by sera derived from LSDV/India/2019 and vice versa, and therefore, Lumpi-ProVac^Ind^ can be considered for use to replace the existing goat pox vaccination practice against LSD in cattle in India. Since the Kenyan-type LSDV strain is predominantly circulating in South Asia, the Lumpi-ProVac^Ind^ could prove to be a better option for the control and eradication of LSD in the region. Commercial production of this vaccine will soon start that will benefit the cattle industry as well as the livelihood of many farmers in the Indian subcontinent.
